# Understanding otherness—the anthropocentrism trap

**DOI:** 10.1007/s00709-025-02043-3

**Published:** 2025-02-13

**Authors:** Peter Nick

**Affiliations:** Joseph Gottlieb Kölreuter Institute for Plant Sciences, Karlsruhe Institute of Technology, Karlsruhe, Germany

For centuries, the position of humans as rulers of our planet has been out of dispute. This viewpoint was fundamentally challenged by the concept of phylogenetic descendance, where humans found themselves to appear as a late step of a gradual process. It seems that Darwin was well aware of the headwind, he would face by spelling this out, because in the Origin of Species, he confined this aspect to a short passage in the conclusion at the very end of the book (Darwin 1859): “*In the distant future I see open fields for far more important researches. Psychology will be based on a new foundation, that of the necessary acquirement of each mental power and capacity by gradation. Light will be thrown on the origin of man and his history.*” After the public had swallowed the fact that we derive from other life forms just like everybody else, at least there remained the consolation that *Homo sapiens* was sketched at the tip of everything else, wide above the lower tiers of life. In his popular seminar for the general public, Ernst Häckel (1874) coined his iconic tree that has shaped the general view on evolution over many decades.

While these anthropocentric visualizations of phylogeny are questioned on occasion (for a critical discussion, see Torrens and Barahona 2012), the concept they transport has remained active. We tend to interpret along lines that are familiar to us. This works relatively neatly, as long as we consider life forms that are close to our own, because, here, we can move along a smooth gradient of progressive distance. Such gradients help to spot homologies. They also safeguard against mistaking apparent similarity as relatedness.

But how to deal with life forms that are obviously fundamentally different? Can we really grasp their otherness at all? There are two traps, where we can step in—either we misinterpret apparent similarities as relatedness, forcing concepts working for us (and life forms very close to us) upon life forms that are essentially different; or we fail to recognize essential congruence because it is manifested in a manner that is unfamiliar to us. One example to study these traps is the debate about plant intelligence or neurobiology (in fact, although often discussed in one breath, these terms stand for congruent, but not identical viewpoints). Some years ago, this journal provided a forum for both parties to explain their standpoint (summaries are given in Nick 2021a, b), first, regarding the question, whether plants are capable of pain (Draguhn et al. 2021, say: no; Baluška and Yokawa 2021, say: yes); second, regarding the question, whether they are conscious and intelligent (Mallat et al., 2021, say: no; Trewavas 2021, says: yes). In that round, both parties had mainly used empirical evidence from the literature to make their point. In the current issue, four years later, we have the chance to watch, how the discussion has evolved since. Has it been enriched by data from new experiments that have been designed to test implications from the hypothesis that plants are or are not intelligent? The answer is a clear no. As one can see from several contributions to the current issue, the debate has shifted to some kind of meta-level, where the two parties mainly talk about terminology, debating strategies, and the history of this debate, only rarely about experiments, data, and phenomena. Nevertheless, in the following, the gross lines of the current discourse will be sketched down to arrive at a short description of its blind spots and suggestions for experimental strategies to fill these spots in the future.

The discussion is opened by the contribution of Kingsland and Taiz (2025). Their main targets are argumentative patterns of plant-neurobiology proponents. They show how quotations from revered historical sources are used to support the case that plants are endowed with intelligence, and they point out that these quotations are subject to confirmation bias. To support their argument, they compare the original sources and their context to the way, how these quotations were paraphrased or integrated. This may appear like a court case with numerous details that are possibly not overly interesting to outsiders of the debate, while it is clear on the other hand that a court case needs to talk about the details. Beyond the general point that science is based on a system of references (either to that what others observed or said, or that what was observed in own experiments) and that precision in referencing is a crucial point for the quality of science, there is a second aspect of general interest: the authors describe the historical context of plant intelligence and also draw interesting links to developments in society that have resonated with the question of sentient plants. They touch different aspects, starting from the relationship between the German Naturphilosophie and the rise of Reizphysiologie (literally irritation physiology) of plants in the late nineteenth century, the controversy around Bose’s experiments, to the postmodern echo of plant sentience in both esoteric and ecological viewpoints.

The charge that the proponents of plant intelligence have quoted and used historical sources incorrectly evokes protest, of course, and it is a matter of fairness that the attacked get a chance to respond. Therefore, this issue also contains the rebuttals to Kingsland and Taiz (2025). The start is made by Calvo et al. (2024), who were under direct attack. While they acknowledge that the use of scientific authorities to support a position is prone to fallacy—in a scientific debate, an argument should stand for itself, no matter, who uttered it (at least according to the Theory of Science), and also can take the point that more empirical evidence is needed, they give examples for confirmation bias on the side of Kingsland and Taiz (2025) themselves. They structure their rebuttal according to the so-called Rapoport Rules (1960) formulated for fruitful handling of dissense—these rules are basically trying to search for common ground, before discussing the points of divergence. While noble in the attitude, the question remains, whether there is more common ground than the call for empirical evidence (“#2. *The evidence, the evidence, and nothing but the evidence*.”). A counter-reply to this rebuttal is given in Kingsland (2025), mainly addressing a quotation of a psychologist, Tolman, that intelligence requires interdisciplinary research. Since this quote was used in the context of human intelligence, they use this as further case of inappropriate use of references. The rebuttal by Minorsky (2024) mainly deals with the campaign of Daniel MacDougal against Chandra Bose in the 1920ies. Bose’s pioneering experiments in plant electrophysiology, widely acknowledged initially, were later refuted as charlatanism in consequence of these attacks that were unfair and, as shown by Minorsky in his previous work, driven by outspoken racism (Minorsky 2021). The motivation for the rebuttal is a passage in Kingsland and Taiz (2025), where the debate around Bose is summarized with the conclusion that his fall was not due to racism, but to a lack of evidence. Moreover, doubt is casted on Minorsky’s scholar attitude. The argument around Bose seems historical at first glance. However, Bose is seen as “father of plant neurobiology” (Minorsky 2021), and, thus, the different narratives on MacDougal’s campaign allude to the current debate on plant neurobiology. The central issue in Minorsky (2024) are charges of confirmation bias against Kingsland and Taiz (2025) and this is the reason for the biblical reference in the title (“*… why beholdest thou the mote that is in thy brother’s eye, but considerest not the beam that is in thine own eye*?).

The rebuttal by Trewavas (2025), who is also explicitly targeted in Kingsland and Taiz (2025), moves along a different line. Contrasting with Calvo et al. (2024), the author does not try to go for a Rapoport approach, but directly rejects the claims made by Kingsland and Taiz, and walks through the individual charges explaining, why, to his opinion, these charges are not appropriate, finishing with a critical look at the group of scientists attacking plant neurobiology. Beyond the mere rebuttal, he also works through a row of evidential arguments in favor of plant intelligence (actually, his is the only contribution in the current battle that refers to empirical evidence, rather than to the statements of the opposing party), also describing, how he was initially intrigued by the discovery of signatures, when it became possible to follow calcium in plants expressing an aequorin transporter. These signatures are qualitatively different depending on the type of challenge posed to the plant (Knight et al. 1991). He also makes an interesting link between the way, how we see plants and the way, how we refer to them. Is respect to other life forms bound to their resemblance to humans? The same author draws a clear distinction between plant neurobiology and plant intelligence: “Nervous systems and brains are not necessary for intelligent behavior, but neural networks almost certainly are.”, a statement that sounds paradox. However, the term neural network is used here in the way as it was originally coined by McCulloch and Pitts (1943) to describe mathematical systems able to match an output to a given input by adjusting the parameters of an intermediate layer (basically the forerunner of that what is nowadays called artificial intelligence). This example touches also a central problem in the entire debate: To describe phenomena that, in their very essence, are inherently different from human experience, terminology is used that has been coined for a different purpose. By the way, the same problem concerns also the public discussion about so-called Artificial Intelligence. To use the same name for different things will cause confusion, if the terminology is not explicitly defined in the very beginning of a contribution. This is rarely done in the debate on plant intelligence, although one needs to give credit to Trewavas (2025) that this author at least provides a definition of what he understands under the term intelligence.

What does this dispute teach us about plants? Very little, unfortunately. We learn more about the ambiguities of human communication. It seems that this discussion is stuck and does not lead somewhere. To get it move on into a more useful direction, two suggestions at the end: 1. Nomenclature: the debate suffers from ambiguous terminology. When the term neural is used outside of metazoan neurons, it becomes misleading—the fact that the term of neural networks had been coined by some mathematicians as metaphor to describe logical networks is already misleading enough. It does not help transparency to use it, for instance, to describe the admittedly dynamic and complex interactions of events in plant signalling. Images have a strong power, they can help us to find explanations, but they can also mislead us to forget about the difference between model and reality. A minimal requirement would be that every participant starts off with definitions, how the terms should be understood. Alternatively, one might consider avoiding nominalisms and rather use verbal language to describe what plants do. Their activities remain complex and wonderful, and they maintain their dignity even if we do not describe them in anthropomorphic language. 2. Experimental design: most experimental evidence used in this debate does not derive from experiments that had been designed to test for intelligence or behavior, but was observed as, often unexpected, side phenomenon. While I concur with Chamovitz (2018) that intelligence is highly subjective, I do not agree that it is, therefore, outside of the realm of science. At least it is possible to operationalize conditions that are needed for intelligence, for instance, learning from previous experience, anticipate future challenges, or adjust responses to complex and contradictive input from the environment. It is also possible to design experiments testing these operationalizations (which includes the possibility of their falsification).

However, the subjective side of plants will remain hidden to us, even if experiments validate such criteria. The same holds true for any subjectivities, though. We can only infer them based on our own subjectivity. This inference gets progressively difficult, the less we can rely on likeness with ourselves. Therefore, we have to face it: we are inevitably trapped in our own anthropocentrism. When we accept this, we might at least arrive in a pragmatic “as if” approach that may open us new insights into the wonders of the plant world.

## References

[CR1] Baluška F, Yokawa K (2021) Anaesthetics and plants: from sensory systems to cognition-based adaptive behaviour. Protoplasma 258:449–454 33462719 10.1007/s00709-020-01594-x PMC7907011

[CR2] Calvo P, Raja V, Segundo-Ortin M (2024) Don’t jump the gun quite yet: aiming for the true target in plant neurobiology research. Protoplasma. 10.1007/s00709-024-01993-4 39340658 10.1007/s00709-024-01993-4 PMC11839687

[CR3] Chamovitz DA (2018) Plants are intelligent; now what? Nat Plants 4:622–623 30185976 10.1038/s41477-018-0237-3

[CR4] Darwin CR (1859) On the origin of species by means of natural selection, or the preservation of favoured races in the struggle for life. John Murray, London, p 488 PMC5184128 30164232

[CR5] Draguhn A, Mallatt JM, Robinson DG (2021) Anesthetics and plants: no pain, no brain, and therefore no consciousness. Protoplasma 258:239–248 32880005 10.1007/s00709-020-01550-9 PMC7907021

[CR6] Häckel E (1874) Natürliche Schöpfungsgeschichte. Gemeinverständliche wissenschaftliche Vorträge über die Entwickelungslehre im Allgemeinen und diejenige von Darwin, Goethe und Lamarck im Besonderen. G. Reimer, Berlin

[CR7] Kingsland SE (2025) Reply to Calvo, Raja, and Segundo-Ortin, “Don’t jump the gun quite yet: aiming for the true target in plant neurobiology research.” Protoplasma. 10.1007/s00709-024-02007-z 10.1007/s00709-024-02007-z 39545947

[CR8] Kingsland SE, Taiz L (2025) Plant “intelligence” and the misuse of historical sources as evidence. Protoplasma. 10.1007/s00709-024-01988-1 10.1007/s00709-024-01988-1 39276228

[CR9] Knight MR, Campbell AK, Smith SM, Trewavas AJ (1991) Transgenic plant aequorin reports the effects of touch and cold-shock and elicitors on cytoplasmic calcium. Nature 352:524–526 1865907 10.1038/352524a0

[CR10] Mallatt J, Blatt MR, Draguhn A, Robinson DG, Taiz L (2021) Debunking a myth: plant consciousness. Protoplasma 258:459–476 33196907 10.1007/s00709-020-01579-w PMC8052213

[CR11] McCulloch W, Pitts W (1943) A logical calculus of ideas immanent in nervous activity. Bull Math Biophys 5:115–133 2185863

[CR12] Minorsky PV (2021) American racism and the lost legacy of Sir Jagadis Chandra Bose, the father of plant neurobiology. Plant Signal Behav 16:1818030 33275072 10.1080/15592324.2020.1818030 PMC7781790

[CR13] Minorsky PV (2024) Matthew 7:3-a response to Kingsland and Taiz (2024). Protoplasma. 10.1007/s00709-024-02002-4 39560739 10.1007/s00709-024-02002-4 PMC11839804

[CR14] Nick P (2021) Intelligence without neurons: a Turing test for plants? Protoplasma 258:455–458 33837845 10.1007/s00709-021-01642-0 PMC8052224

[CR15] Nick P (2021) Sensitive or sentient—a painful debate. Protoplasma 258:235–238 33580839 10.1007/s00709-021-01621-5 PMC7907014

[CR16] Rapoport A (1960) Fights, games, and debates. University of Michigan Press, Ann Arbor

[CR17] Torrens E, Barahona A (2012) Why are some evolutionary trees in natural history museums prone to being misinterpreted? Evo Edu Outreach 5:76–100

[CR18] Trewavas A (2021) Awareness and integrated information theory identify plant meristems as sites of conscious activity. Protoplasma 258:673–679 33745091 10.1007/s00709-021-01633-1 PMC8052216

[CR19] Trewavas A (2025) Plant intelligence dux: a comprehensive rebuttal of Kingsland and Taiz. Protoplasma. 10.1007/s00709-024-02005-1 10.1007/s00709-024-02005-1 PMC11839692 39505772

